# Study on the Improvement of Droplet Penetration Effect by Nozzle Tilt Angle under the Influence of Orthogonal Side Wind

**DOI:** 10.3390/s24092685

**Published:** 2024-04-24

**Authors:** Daozong Sun, Junyutai Hu, Xinghan Huang, Wenhao Luo, Shuran Song, Xiuyun Xue

**Affiliations:** 1 College of Electronic Engineering (College of Artificial Intelligence), South China Agricultural University, Guangzhou 510642, China; 2 College of Engineering, South China Agricultural University, Guangzhou 510642, China; 3 Guangdong Engineering Research Center for Monitoring Agricultural Information, Guangzhou 510642, China

**Keywords:** wind speed, nozzle tilt angle, spraying, droplet penetration

## Abstract

This study investigates the impact of varying side wind velocities and nozzle inclination angles on droplet penetration during plant protection spraying operations, focusing on citrus trees. Experiments were conducted across four wind speed levels (0, 1, 2, 3 m/s) perpendicular to the nozzle direction and seven nozzle inclination levels (0°, 8°, 15°, 23°, 30°, 38°, 45°) to evaluate droplet distribution under different spraying parameters. A baseline condition with 0 m/s wind speed and a 0° nozzle angle served as the control. Utilizing Computational Fluid Dynamics (CFD) and regression analysis techniques in conjunction with field trials, the droplet penetration was analyzed. Results indicate that at constant wind speeds, adjusting the nozzle inclination angle against the direction of the side wind can significantly enhance droplet deposition in the canopy, with a 23° inclination providing the optimal increase in deposition volume, averaging a change of +16.705 μL/cm^2^. Multivariate nonlinear regression analysis revealed that both wind speed and nozzle inclination angle significantly affect the droplet penetration ratio, demonstrating a correlation between these factors, with wind speed exerting a greater impact than nozzle angle. Increasing the nozzle inclination angle at higher wind speeds improves the penetration ratio, with the optimal parameters being a 23° angle and 3 m/s wind speed, showing a 12.6% improvement over the control. The model fitted for the impact of nozzle angle and wind speed on droplet penetration was validated through field experiments, identifying optimal angles for enhancing penetration at wind speeds of 1, 2, and 3 m/s as 8°, 17°, and 25°, respectively. This research provides insights for improving droplet penetration techniques in plant protection operations.

## 1. Introduction

Uniform droplet deposition within the crop canopy is fundamental for precision application, significantly influencing the quality of pesticide application [1]. Accumulation of droplets, hindered by the dense outer leaf canopy, often results in runoff losses, compromising effective pest and disease control within the canopy and on the trunk [2]. Overcoming environmental barriers to ensure effective droplet penetration across the canopy is critical for achieving uniform deposition. Challenges such as droplet deposition attenuation and uneven leaf surface adhesion arise from non-uniform penetration distributions within canopies with high closure [3]. Therefore, enhancing droplet penetration is paramount for improving spray outcomes [4]. Several factors, including canopy structure [5], spray parameters [6], and airflow dynamics [7,8], affect droplet penetration. Studies by Duga [9] et al. on different canopy structures have shown that tree volume, canopy density, and leaf wall porosity significantly influence droplet deposition rates and penetration capabilities. Foqué and Nuyttens [10] suggested that exploring the combined effects of spray angle, airflow, and spray volume could further enhance crop penetration and deposition effects. Moreover, Ru [11] et al. developed a model for droplet penetration rates based on wind tunnel speed, porosity, and collection point depth by investigating the relationships within the fruit tree canopy.

Technological advancements, such as airflow-assisted spraying [12,13], electrostatic spraying [14,15], boom spraying [16], and contour spraying [17], have been implemented to improve droplet penetration in agricultural production. Traditional boom sprayers, prevalent in field crops, rely on adjustments to spray pressure, flow rate, and nozzle types to enhance penetration, which may also impact spray outcomes [18]. Modifying the nozzle’s inclination angle has proven effective for improving deposition effects [19,20,21], though research has primarily focused on vertical spraying. Less attention has been given to the effects of wind speed and nozzle inclination on droplet penetration under horizontal spraying, commonly used in fruit tree protection operations.

This study investigates the relationship between the tilt angle of spray nozzles and the penetration ratio of droplets under crosswind conditions, employing a controlled wind field to simulate variable wind speeds. Through the adjustment of the nozzle’s tilt angle, it utilizes Computational Fluid Dynamics (CFD) and multivariate nonlinear regression analysis, augmented by field experiments, to systematically assess the influence of crosswind velocity and nozzle inclination on droplet penetration. The objective is to elucidate patterns that will facilitate the optimization of operational parameters for pesticide application equipment, enabling precise pesticide delivery and enhancing the efficacy of pest and disease management in orchards.

## 2. Materials and Methods

### 2.1. Test Materials

**Figure 1:**
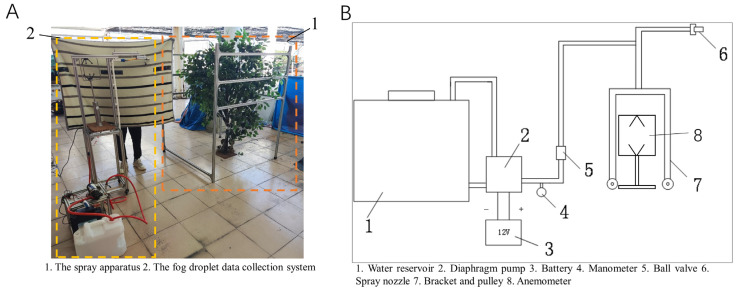
(**A**) Test site. (**B**) Structure of the spraying device.

**Figure 2:**
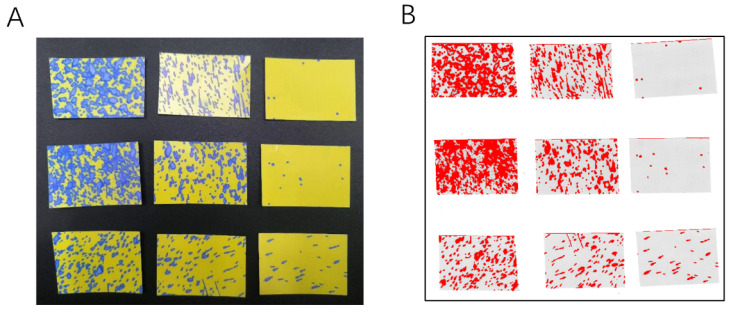
(**A**) Sample point Scan image. (**B**) Processed images.

The spray trial system primarily comprises two main components, as illustrated in Figure 1A: the spraying device and the droplet information collection system. The droplet information collection system, labeled as number 1 in Figure 1A, primarily consists of an aluminum frame and a simulated tree. The frame features three horizontal beams on each side at heights of 90 cm, 120 cm, and 150 cm, respectively. These beams are connected through the simulated tree using capillary threads, with water-sensitive papers mounted on these threads to collect droplet information [22]. The frame’s dimensions are 120 cm × 120 cm × 180 cm, and the water-sensitive papers are 76 mm × 26 mm in size. DepositScan (DepositScan v1.2, USDA-ARS Application Technology Research Unit, Wooster, OH, USA), the imaging software used, can quantify the distribution of spray deposits on any paper-type collector by enhancing the visual contrast between the spray deposits and the background. It also analyzes the droplet parameter information on the collectors [23]. The distribution of droplets on the water-sensitive papers is an approximate representation of the liquid distribution on leaves. Figure 2 shows scanned images of water-sensitive papers from selected sampling points at various depths alongside the image processing interface.

The actual spraying device, depicted in item 2 of Figure 1A and its detailed structure in Figure 1B, includes a water tank, a diaphragm pump, a battery, a pressure gauge, switches, and an anemometer. The battery is a 12 V lead-acid type; the diaphragm pump, model DP-160, has a flow rate of 7 L/min and a pressure range of up to 0.8 Mpa; the pressure gauge, model PTB203S, ranges up to 3 Mpa; the nozzle is a JJXP model standard solid cone type, rated at 0.2 Mpa pressure; and the anemometer, model WindMaster Pro (WindMaster Pro, Gill Instruments Ltd., Lymington, Hampshire, UK), can measure three-dimensional wind speed and direction, ranging from 0 to 65 m/s.

The simulated tree was designed to mimic the common specifications of citrus trees used in our field trials, with a height of 180 cm, a canopy width of 150 cm, a designed volume of 1,413,716.69 cm^3^, a projected area of 17,671.46 cm^2^, 108 branches, a leaf area index of 5.48, and a canopy density consistent in all directions. The field trial involved three consecutively arranged citrus trees in an orchard, each with an average height of 180 cm, uniform canopy density, and spaced 150 cm apart, aligned in a north-south direction.

### 2.2. Experimental Design and Methods

#### 2.2.1. Determination of Test Parameters

The assessment of droplet penetration distribution was quantified through two metrics: penetration deposition and penetration ratio. Penetration deposition quantifies the depth-wise deposition of droplets within the canopy, with the mean droplet deposition on each vertical plane representing this measure [3]. This measure is further detailed by calculating the confidence interval for the mean deposition quantity, illustrating the variability of sampling points across the specified vertical plane. Comparative analysis used the change in deposition value to reflect the experimental group’s deposition variance from the control. The average deposition quantity is calculated using Equation (1):

(1) $$Q_{i}=\frac{1}{9}\sum_{j=1}^{9}q_{ij}$$

where:

*i*: Position of the droplet distribution layer. In this experiment, three layers were set up, *i* = 0, 1, and 2, where *i* equals 0 for the outermost layer,

*j*: Position index of the droplet capture points on each layer’s capture surface, ranging from 1 to 9, denoting top to bottom and left to right orientation,

q*_ij_*: Deposition quantity at capture points, measured by DepositScan software analysis on water-sensitive paper.

The change in deposition is given by Equation (2):

(2) $$D_{i}^{vn}=Q_{i}^{vn}-Q_{i}^{v0}$$

where:

*v*: Wind speed value (0, 1, 2, 3),

*n*: Nozzle tilt angle (0°, 8°, 15°, 23°, 30°, 38°, 45°).

The droplet penetration ratio is articulated as the percentage of droplet penetration, which facilitates the quantitative evaluation of droplet penetration efficacy within the canopy. It is defined as the ratio of the average droplet volume penetrating a canopy unit on a vertical plane to that just before the canopy edge. This is expressed by Equation (3):

(3) $$P_{i}=\frac{Q_{i}}{Q_{0}}\times 100\%$$

where:

*i*: Droplet distribution layer index (1, 2),

*Q_i_*: Mean droplet volume on the vertical surface corresponding to canopy unit i, in μL/cm^2^,

*Q*_0_: Mean droplet volume on the vertical surface of the unit just outside the canopy edge, in μL/cm^2^,

*P_i_*: Penetration ratio of droplets for layer i, expressed as a percentage.

The formula for calculating the confidence interval (*CI*) is given by Equation (4):

(4) $$CI=\bar{x}\pm (t_{\frac{\alpha }{2},df}\times \frac{s}{\sqrt{n}})$$

where:

*CI*: Confidence interval,

$\bar{x}$: Sample mean,

$t_{\frac{\alpha }{2},df}$: Critical value from the t-distribution, aligned with the selected confidence level and degrees of freedom. A 95% confidence level is assumed in this context,

*s*: Sample standard deviation,

*n*: Sample size.

**Figure 3:**
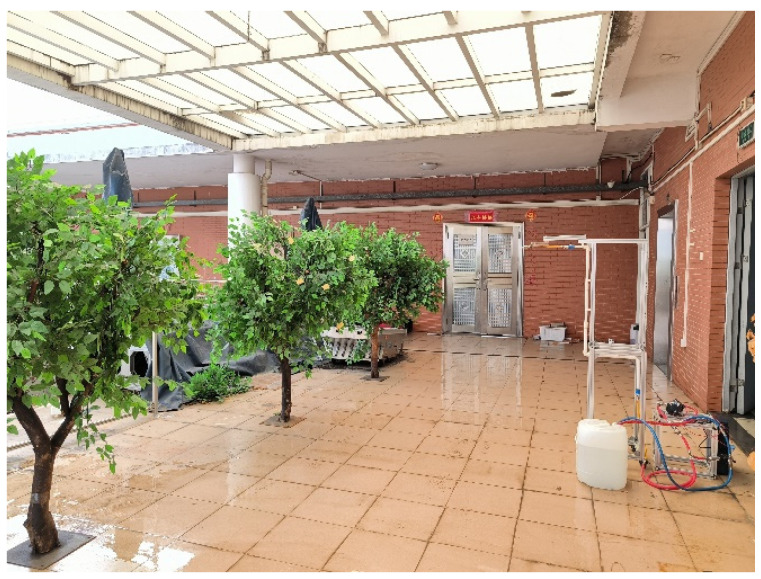
Pre-experimental site.

Given that trees are distributed continuously under actual field conditions, a preliminary experiment was designed to isolate the potential influence of surrounding trees on the subject of this study. As illustrated in Figure 3, the spraying device was used to apply treatments to both a subject with no surrounding trees, designated as the control group and a subject surrounded by other trees, designated as the experimental group. The inter-tree spacing for the simulated trees was set at 150 cm, with a spraying distance of 120 cm, side wind velocity at 1 m/s, and a nozzle deflection angle of 15°, aligning with the actual field conditions. Nine water-sensitive papers were uniformly distributed at the outer, middle, and inner layers of the canopy to record deposition patterns. The droplet penetration ratio was calculated using Equation (3). The experiment was conducted three times, and the results were averaged.

**Figure 4:**
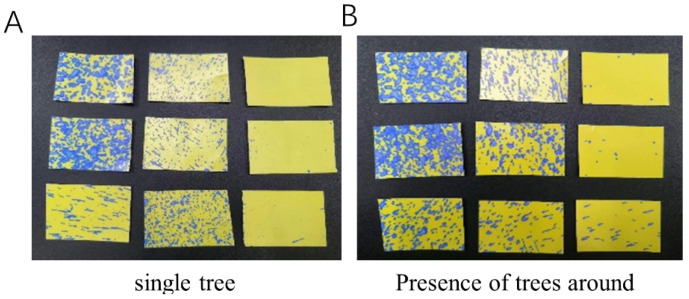
Comparison of droplet deposition for the test. (**A**) shows the case of a single tree; (**B**) shows the case of the presence of other trees in the surrounding area.

Experimental results, as shown in Table 1, indicate the significance of *p*-values as defined by Equation (3): P1 represents the penetration ratio at the front of the canopy, P2 denotes the penetration ratio within the canopy, and P signifies the average penetration ratio across the canopy. The data reveal that the penetration ratio of the first layer of droplets in the experimental group is slightly lower than that in the control group, whereas the second layer’s penetration ratio exceeds that of the control. This discrepancy can be attributed to the increased susceptibility of individual trees to environmental influences compared to those surrounded by other trees. In the absence of neighboring trees to obstruct side winds, individual trees are exposed to stronger lateral wind effects, resulting in finer droplets on the canopy surface and, consequently, reduced deposition volumes, as illustrated in Figure 4. Additionally, this situation intensifies the internal air turbulence within the canopy, enhancing the airflow’s capacity to carry droplets and thereby increasing the penetration ratio. Overall, nearby trees impact the subject of the experiment, decreasing the droplet penetration ratio compared to areas devoid of surrounding trees. Droplet deposition patterns, as depicted in Figure 4, are similar across experiments, with droplets primarily distributed on the left side’s water-sensitive paper. The smaller droplet size in the case of individual trees may result from the surrounding trees partially obstructing the wind field, diminishing the environmental wind’s impact on the target. The difference is negligible, with a decrease of approximately 0.01, leading to the exclusion of surrounding trees’ influence in this experiment and the selection of an individual tree for testing. Further research will delve into the environmental impacts on the target tree.

**Figure 5:**
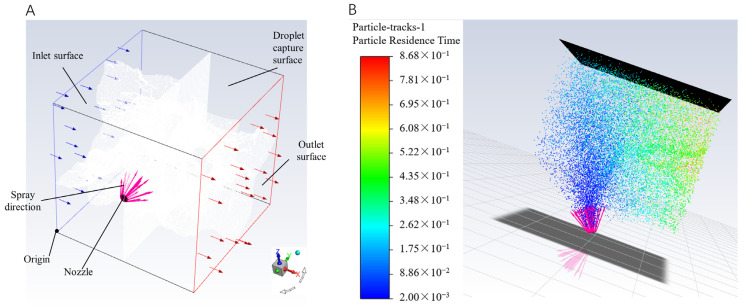
(**A**) Calculation field for spray range; (**B**) Simulation of spraying process.

This study refers to the approach proposed by Sun [18] et al. and employs Computational Fluid Dynamics (CFD) simulations using Ansys Fluent 21.1.0 to determine the range of nozzle tilt angles and side wind velocities based on experimental conditions. To accurately simulate the spraying process under real conditions, the computational domain dimensions and boundary conditions from the nozzle to the canopy were meticulously defined. The spraying distance was set at 120 cm, with the target canopy’s dimensions approximately 120 cm in length, width, and height. The nozzle was positioned at the center of the canopy, with the side wind direction perpendicular to the nozzle direction. Consequently, the computational domain for the simulation, as illustrated in Figure 5A, was designed with dimensions of 120 cm in length, width, and height, placing the nozzle at coordinates Z = 60 cm, X = 60 cm, Y = 0 cm, with the initial spraying direction toward +Y. Gravity was set in the −Z direction at 9.81 m/s^2^. The side wind turbulence was modeled using the standard k-epsilon viscosity model [24]. Spray parameters, including the average droplet diameter of 270 μm, spray cone angle of 30°, and velocity of 20 m/s, were measured using a laboratory particle dynamics analyzer based on actual experimental parameters. A discrete phase model was selected, setting the spray source type as solid cone, treating the spray as a discrete phase, and the side wind airflow as a continuous phase. Given the focus on canopy droplet distribution, the Discrete Phase Model (DPM) boundary condition for the canopy capture surface was set to ‘trap’ to represent droplet capture, recorded on the capture surface, while other wall DPM conditions were set to ‘escape’, indicating droplet escape without capture. Adjustments to side wind speed and source angle were made during simulations to observe droplet distribution on the capture surface and determine spray effectiveness. Particle tracking trajectories during the spray process are shown in Figure 5B.

**Figure 6:**
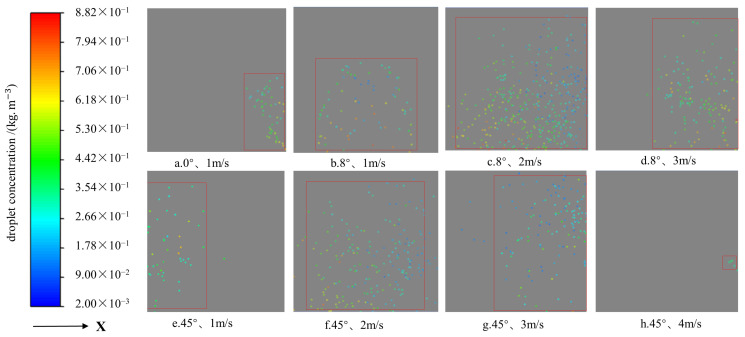
Droplet distribution on the droplet capture surface for different parameters. Note: The rectangular box in the figure shows the extent of the droplet distribution.

Under various combinations of side wind speeds, nozzle inclination angles, and source tilt angles, the distribution of droplets on the capture surface is detailed in Figure 6. With a 0° inclination angle and a side wind speed of 1 m/s, droplets were significantly displaced by the wind, predominantly at the capture surface’s edge. As wind speed increased, the droplet distribution on the capture surface shifted toward the +X direction, increasing the quantity of captured droplets. Notably, adjusting the nozzle inclination angle to approximately 8° under side wind conditions allowed for a broader area of droplet capture. With further increases in the inclination angle, droplet distribution moved toward the −X direction, with a decrease in droplet quantity. At a 45° angle, droplets were primarily concentrated at the capture surface’s left edge, and beyond this angle, droplet capture was nearly unsuccessful. Consequently, the experimental conditions were set with nozzle inclination angles ranging from 8° to 45° and wind speed levels from 1 to 3 m/s based on a comprehensive analysis.

#### 2.2.2. Spray Test Design

This study investigates the effect of different nozzle inclination angles on droplet penetration under various side wind speeds. Experiments were conducted at a nozzle height of 120 cm above the ground and a spray pressure of 0.3 MPa. Four levels of wind speed (0, 1, 2, 3 m/s) and seven nozzle inclination angles (0°, 8°, 15°, 23°, 30°, 38°, 45°) were tested, with the 0 m/s wind speed and 0° inclination angle serving as the control group.

**Figure 7:**
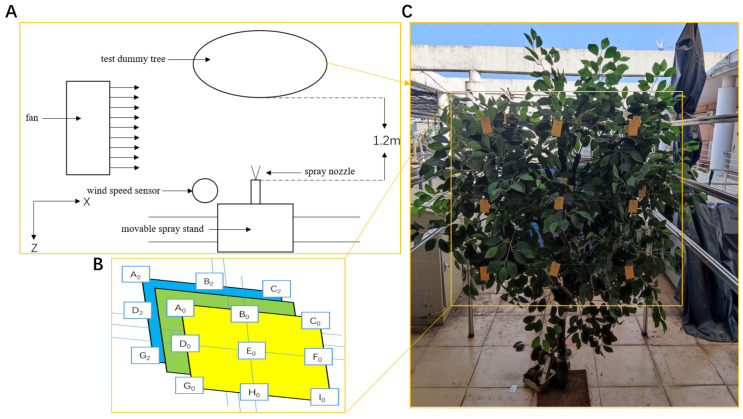
(**A**) Structure of droplet penetration test; (**B**) Schematic diagram of the location of measurement points; (**C**) Sampling point layout.

The experimental setup, as depicted in Figure 7A, features a nozzle mounted on a stand with an initial spray direction perpendicular to the artificial tree, allowing for adjustable spray angles. A fan generates a constant wind field in the +X direction, perpendicular to the initial spray direction, with an anemometer placed 50 cm in the −X direction from the nozzle. The artificial tree is positioned 120 cm perpendicular to the spraying device. Water was used as a surrogate for pesticide in the spray liquid, with the nozzle inclination adjusted from −Z to −X.

Droplet collection sampling points within the canopy were arranged as follows: the canopy was divided into three layers from front to back along the spray direction, with each layer spaced 20–25 cm apart. Nine measurement points were positioned on each layer (A_i_, B_i_, C_i_, …, I_i_), with distances of 30–35 cm between A_i_, B_i_, and C_i_, and a 30 cm interval between A_i_, D_i_, and G_i_, as illustrated in Figure 7B.

The method for measuring the droplet penetration ratio is outlined below:

1. As illustrated in Figure 7C, the canopy is divided into three equal layers from front to back along the spray direction. On each side of the layer, three horizontal beams support fine lines threaded through the canopy in a straight line, with nine measurement points (A_i_, B_i_, C_i_, …, I_i_) evenly arranged on the plane formed by the three lines. Water-sensitive papers are attached at each point with paper clips positioned perpendicular to the spray direction.
2. In order to prevent obstruction between front and back water-sensitive papers, slight adjustments are made to their positions. If foliage obstructs the lines, they are adjusted to ensure a straight path through the canopy, maintaining all three lines in the same vertical plane.
3. At each wind speed and nozzle inclination angle, a one-second spray operation is conducted. After the water-sensitive papers dry, they are collected in sequence into a storage box and labeled according to the order of the experiment. Each experimental condition is repeated three times, with results averaged.
4. Wind speed and nozzle inclination angles are varied, repeating step 3 until droplet sampling for all conditions is completed.
5. DepositScan software is used to scan and analyze the deposition on water-sensitive papers.
6. The average of the parameters from all sampling points on the same layer represents the average parameter on that vertical cross-section of the canopy. Equations (1) and (3) are used to calculate the droplet penetration ratio for each layer.

#### 2.2.3. Field Trial Design

**Figure 8:**
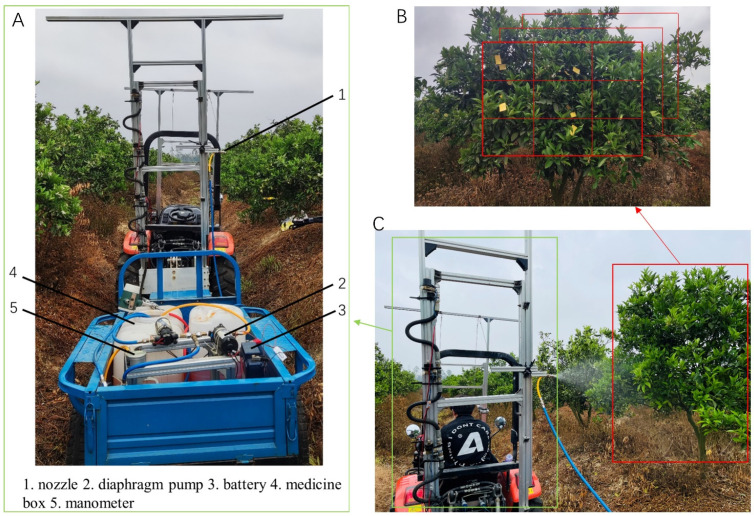
(**A**) General structure of the field test sprayer; (**B**) Layout of sampling points for field trials; (**C**) Schematic diagram of spraying operation.

A field trial was designed to validate the droplet penetration patterns observed in the spray experiment under real-field conditions. Given the uncontrollable nature of natural wind speeds in the field, the trials were conducted in the absence of natural wind, employing varying vehicle speeds to simulate side wind effects. The field trial sprayer setup, illustrated in Figure 8A, closely mirrors the laboratory spraying apparatus. It is mounted on an aluminum mast attached to the rear of a tractor. The nozzle, positioned 1.2 m above the ground and 1.2 m from the target citrus canopy, dispenses the spray liquid pumped from a tank by a diaphragm pump through hoses, operating at a pressure of 0.3 MPa.

Three adjacent citrus trees of similar size were selected for the experiment. The canopy of each tree was divided into three layers, spaced 20–25 cm apart. Similar to Figure 7B, nine water-sensitive papers were evenly arranged in each layer, with vertical and horizontal spacings of 25–30 cm, totaling 27 papers per tree and 81 papers across all three trees. Due to field trial constraints, water-sensitive papers were directly placed on the leaves, as depicted in Figure 8B. Pure water was set up as a spray solution, and the nozzle inclination was set to 0°. A static spray was applied for 1 s as the sprayer passed each target citrus tree, as demonstrated in Figure 8C. After the papers dried, they were collected from left to right, top to bottom, and front to back into a sealed dry box to prevent contamination from handling (all handling was undertaken wearing disposable rubber gloves to avoid image analysis interference from hand sweat). Subsequent sets of water-sensitive papers were then arranged for the next group of experiments.

Based on conclusions from spray tests (Section 3.1.2), nozzle inclination angles were set to 0°, 8°, 17°, and 25°, aligned with the direction of the sprayer’s movement, perpendicular to the initial nozzle direction. Due to the uncontrolled side wind in field conditions, three different sprayer travel speeds were set relative to the direction of the environmental wind, simulating three distinct external influences. The sum of the side wind speed and vehicle speed represented the relative wind speed. Preliminary experiments determined that setting the sprayer’s travel speed to achieve relative wind speeds of 1, 2, and 3 m/s allowed for an effective spraying duration of approximately 1 s under each of the four inclination angles. Each change in the sprayer’s speed required passing through the target citrus trees for the spraying operation.

## 3. Results

The experiment was conducted using the controlled variable method, with the spray test site on the sixth floor overhead of the North Building of the College of Engineering, South China Agricultural University, Guangdong Province, China, in December 2022, and the field test site at the base of Great Orange Orchard Pingtan, Pingtan Town, Huidong County, Huizhou City, Guangdong Province, China, in March 2023, with an average ambient temperature of 21 °C and average ambient humidity of 56% at the time of the test.

### 3.1. Spray Test Results and Analysis

#### 3.1.1. Distribution of Droplet Penetration Deposits

**Figure 9:**
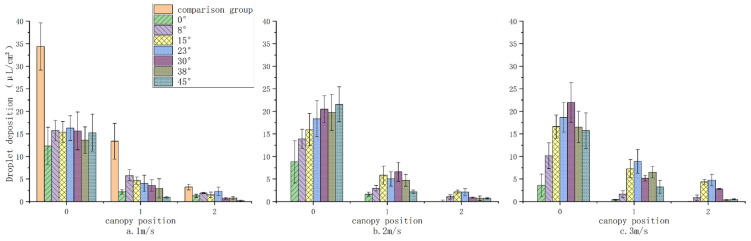
Average deposition on each layer for different parameters. Note: The experimental conditions for the control group in (**a**) were 0° tilt angle, 0 m/s wind speed.

Figure 9 presents a comparison of deposition quantities under varying wind speed conditions across different tilt angles, with the deposition quantity averaged across all layers within each condition, represented with 95% confidence intervals as error bars. The deposition at the outermost layer (level 0) is significantly higher than at the middle (level 1) and innermost layers (level 2). It is observed that the volume of droplets decreases by approximately 60% after traversing the first canopy layer and by about 76% after the second layer. This reduction indicates that obstructions such as branches, leaves, and gravitational forces lead to a considerable decrease in droplet numbers upon canopy penetration, resulting in the majority of droplets depositing on the canopy surface. Under low wind speed conditions (1 m/s), the deposition on the outermost layer initially increases with the nozzle inclination angle before decreasing, reaching a peak value. This indicates the existence of an optimal nozzle inclination angle that maximizes outer canopy deposition. Although the middle and inner layers also show fluctuations in deposition with changes in nozzle inclination, these are not as marked as in the outer layer. In Figure 9a, deposition amounts are similar across all inclination angles. In Figure 9b, deposition gradually increases with the inclination angle, while in Figure 9c, there is an initial increase followed by a decrease in deposition, peaking at a 30° inclination. The observed deposition patterns can be attributed to the following: at a wind speed of 1 m/s, droplet drift is minimal, allowing most droplets to reach the target, thereby minimizing the influence of inclination angle. At 2 m/s, the drift becomes more pronounced, and adjustments in inclination angle can mitigate this drift, having a greater impact on deposition. At 3 m/s, particularly when the inclination angle exceeds 30°, the angle of interaction between the droplets’ initial trajectory and the wind direction significantly influences droplet fragmentation. This leads to larger droplets breaking down into smaller ones, increasing drift due to reduced droplet size, where the effect of drift outweighs the compensatory mechanisms, resulting in decreased deposition [18]. Within the canopy, the deposition distribution typically shows a pattern of increase followed by a decrease, primarily concentrated between 15° and 30° angles. Notably, deposition at a 23° inclination angle is consistently higher, with the highest deposition amounts for both the middle and innermost layers occurring at this angle and at a 3 m/s wind speed. The lowest deposition is recorded at a 45° inclination angle due to the change in spray angle, which reduces the initial velocity of the droplets in the 0° direction, thus diminishing their penetration distance and preventing many droplets from reaching the second layer. The error bars indicate greater variability in deposition quantity on the canopy’s outer side and under conditions of lower tilt angles. This variability could be attributed to the unevenness of droplet deposition caused by side wind effects or inherent measurement errors, as illustrated in Figure 4. Conversely, within the canopy’s inner side and under conditions of higher tilt angles, the variability in deposition quantity is reduced, likely due to generally lower deposition quantities within the canopy. In summary, a nozzle inclination of 23° provides the most significant improvement in droplet penetration and deposition, especially within the canopy. These findings align with the expected droplet deposition patterns in practical field applications.

The control group results, established at a 0° inclination angle and 0 m/s wind speed, demonstrated that the amount of droplet deposition in all layers of the experimental group was smaller than that of the control group, highlighting the substantial impact of wind speed on droplet deposition [25]. The presence of side wind or shifts in nozzle inclination affects droplet trajectories due to horizontal air resistance, causing some droplets to deviate from their intended target [26]. Additionally, the initial direction of droplet movement, when counter to the side wind direction, exacerbates inter-droplet dynamics, leading to increased aggregation, fragmentation, and secondary breakup events [27]. This results in larger droplets fragmenting into smaller counterparts, thereby hindering their ability to reach the target under aerodynamic influences.

Comparing deposition amounts under varying nozzle inclination angles at constant wind speeds with those at a 0° inclination angle elucidates the impact of nozzle adjustment on target deposition under equivalent side wind conditions. Table 2 details the changes in deposition amounts across different vertical planes under various wind speeds and nozzle inclinations. The majority of these changes are positive, indicating that at the same wind speeds, deposition at a 0° inclination angle is generally lower than that at other angles. Furthermore, as indicated by Figure 9, at wind speeds of 2 m/s and 3 m/s, the inner canopy layers exhibit negligible droplet presence under a 0° condition, while a discernible capture of droplets occurs at other angles. This suggests that adjusting the nozzle inclination can mitigate issues of insufficient droplet deposition within the canopy. Horizontally, the data’s magnitude relation generally follow a pattern of D_0_ > D_1_ > D_2_, indicating that the influence of inclination angle on deposition change is constrained by canopy depth, with deeper layers exhibiting smaller changes. Therefore, compared to the control group, altering the nozzle inclination can effectively enhance the overall deposition across the canopy layers, particularly on the outermost layer, and also increase the deposition within the canopy, thus improving the penetration effect of droplets.

The cumulative change in deposition amounts for all three layers under the same experimental conditions represents the overall change for that scenario. The average of the total changes under different side winds for the same inclination angle represents the average change in deposition for that angle. Calculations yield average changes in deposition amounts at inclination angles of 8°, 15°, 23°, 30°, 38°, and 45° as +7.949, +14.493, +16.705, +15.863, +11.905, and +10.031 μL/cm^2^, respectively, with the optimal deposition effect observed at a 23° angle. Specifically, the largest overall change in deposition amount occurs at this angle with a 3 m/s wind speed, reaching a value of 28.284 μL/cm^2^. These findings imply that at constant wind speeds, appropriately adjusting the nozzle inclination against the side wind direction can significantly enhance droplet deposition in the canopy. At a 0° inclination, side wind alters the droplet trajectory, causing most droplets to miss the target. Adjusting the nozzle inclination angle can compensate for droplet drift [18].

#### 3.1.2. Analysis of Droplet Penetration Ratio

**Figure 10:**
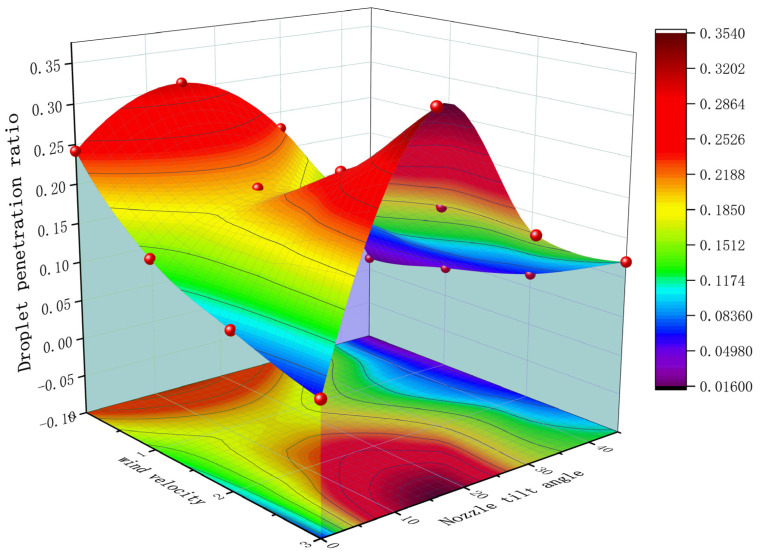
Droplet penetration ratio for different parameters.

Figure 10 illustrates the variation in droplet penetration ratio under different conditions, showing a pattern where the ratio initially rises and then falls with the increase of the nozzle tilt angle across three distinct wind speeds. Specifically, under a 1 m/s wind condition, the optimal penetration ratio occurs at an 8° tilt; at 2 m/s, the peak ratio is found at 15°; and at 3 m/s, the highest ratio is recorded at 23°—the apex across all tested conditions. Remarkably, four experimental setups exceed the control group in penetration ratio, with the most significant difference reaching 12.6%. This demonstrates that adjusting the nozzle tilt angle can effectively improve the droplet penetration ratio under various side wind conditions.

At a 0° tilt angle, side wind causes droplet drift, preventing the majority from reaching or penetrating the target. As wind speed increases, penetration effectiveness correspondingly declines. At an 8° tilt, the average droplet penetration ratio gradually reduces with increasing wind speed, indicating that the wind’s influence on droplet penetration exceeds that of the tilt angle. Beyond an 8° tilt, the average penetration ratio systematically rises with wind speed, suggesting the tilt angle’s effect on penetration becomes more pronounced than that of wind speed. The improvement in droplet penetration ratio is attributed to (1) the propensity for droplets to break into smaller fragments under the combined effect of side wind and nozzle tilt, enabling them to navigate through pores otherwise too small for larger droplets, and (2) the movement of canopy leaves and branches induced by side wind, which changes the canopy’s porosity—opening previously tight gaps and facilitating droplet passage [28]. When the tilt angle is set to 45°, and the wind speed is 1 m/s, the penetration ratio is at its lowest, with most droplets missing the target. However, as wind speed increases, the penetration ratio gradually improves. This enhancement is partly because the higher wind speed redirects stray droplets toward the target and partly due to the change in droplet impact angle on the leaves. In the control group, without side wind, droplets approach the leaf surface nearly perpendicularly, creating a larger area of obstruction. Conversely, with significant tilt angle adjustments under side wind, more droplets enter the canopy laterally rather than directly hitting the leaf surface, facing less obstruction due to the reduced angle of incidence, which promotes deeper canopy penetration.

**Figure 11:**
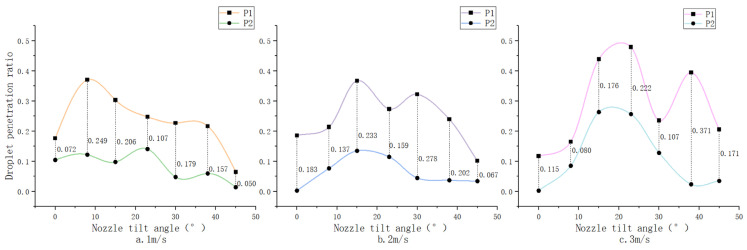
Variation of the penetration ratio of fog droplets for different parameters. Note: P1 denotes the percentage of droplet penetration in layer 1, P2 denotes the percentage of droplet penetration in layer 2, and the numbers represent the differences.

Figure 11 depicts the changes in droplet penetration ratio under various conditions. The difference between P1 and P2 indicates the attenuation effect within the canopy, with larger gaps signifying more significant attenuation. It is observed that the penetration ratio at the first layer surpasses that at the second layer, with both layers displaying similar trends within the margin of error, consistent with previous analyses of average penetration ratio trends. At a 0° nozzle tilt, side wind-induced drift markedly reduces droplet capture within the canopy, causing considerable inconsistency between P1 and P2, rendering it unreliable. Under a 1 m/s wind speed, the disparity lessens, indicating reduced attenuation. At 2 m/s, the difference first enlarges and then contracts, remaining relatively stable except at a 45° tilt, where most droplets miss the target, resulting in negligible droplet presence within the canopy. For a 3 m/s wind speed, the disparity shows variability, with the overall trend being minor compared to other wind conditions. The most pronounced discrepancy between the two layers occurs at a 38° tilt, indicating the most significant attenuation effect within the canopy under this scenario. The interaction between side wind and nozzle tilt angle effectively mitigates droplet attenuation during canopy penetration. This could be due to the dynamic alteration in canopy porosity from static to dynamic in the presence of side wind, enhancing droplet penetration [28]. As the tilt angle increases under side wind, facilitating lateral entry of droplets into the canopy, the difference in penetration ratio significantly diminishes. This results from the altered direction of droplet movement, which substantially reduces impediment by the leaves, thus lessening the attenuation effect within the canopy [29].

**Figure 12:**
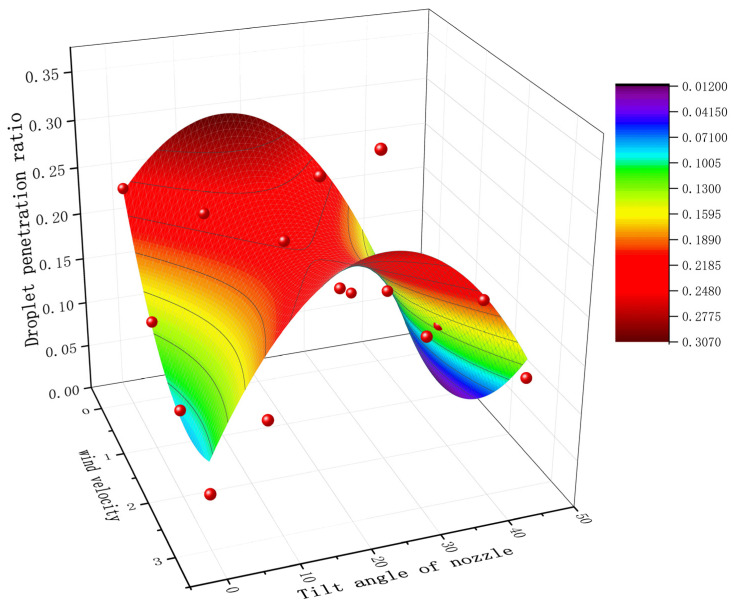
Multivariate nonlinear surface fitting graph.

Figure 12 illustrates the outcome of a multivariate nonlinear regression analysis, employing wind speed and nozzle tilt angle as independent variables and the droplet penetration ratio as the dependent variable. The regression equation, as indicated by Equation (5), is expressed as follows:

(5) $$P=av^{2}+bn^{2}+cvn+dv+en+f$$

where *P* represents the droplet penetration ratio, *v* represents the side wind speed in m/s, and *n* represents the nozzle tilt angle in degrees.

The fitted results, presented alongside Table 3, reveal that the regression model boasts a determination coefficient (R^2^) of 0.8522, indicating an excellent fit and strong correlation. Notably, the coefficient for variable v (wind speed) is higher than that for n (nozzle tilt angle), highlighting the more significant impact of wind speed on droplet penetration compared to the nozzle tilt angle. The interaction term vn within the model captures the synergy between wind speed and nozzle tilt angle, with its positive coefficient suggesting that an increase in nozzle tilt angle can amplify the droplet penetration ratio as wind speed escalates. Upon analysis, the multivariate nonlinear regression model identifies optimal tilt angles for enhancing horizontal droplet penetration ratios at wind speeds of 1, 2, and 3 m/s as 8°, 17°, and 25°, respectively.

In conclusion, modifications in wind speed and nozzle tilt angle can effectively enhance droplet penetration within the canopy. Adjusting the nozzle tilt angle according to different wind speeds can compensate for the droplet drift phenomenon, thereby augmenting both the quantity of droplet deposition and the penetration rate. However, it is observed that the total amount of deposition decreases when compared to spraying vertically without the influence of wind. The identified optimal spraying parameters for maximizing droplet penetration include a 23° tilt angle at a wind speed of 3 m/s. Given the variable nature of wind speed and direction in actual spraying operations and the varying spray requirements for different plant positions [8], careful selection of the spray nozzle tilt angle is imperative.

### 3.2. Field Trial Results and Analysis

**Figure 13:**
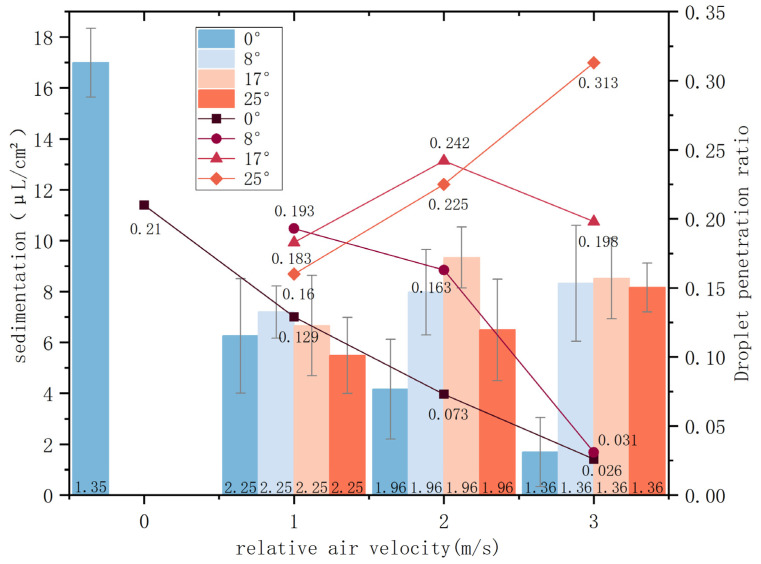
Graph of field test results.

The average deposition amounts and droplet penetration ratios under different nozzle tilt angles and relative wind speeds are illustrated in Figure 13, with error bars representing 95% confidence intervals. The observed trends in the field experiment parameters are consistent with those from laboratory experiments. When utilizing the data from a 0° tilt angle and 0 m/s relative air velocity as a baseline, it was found that deposition under all tested conditions was lower than the baseline, with most droplet penetration ratios also below the baseline group. This reduction is attributed to changes in the initial direction of droplet velocity, external air resistance, and a reduced spray distance, leading to droplet drift and constrained penetration depth. At a constant relative air velocity, deposition amounts initially rise and then fall with increasing tilt angles. The peak values for relative air velocities of 1 m/s, 2 m/s, and 3 m/s were observed at tilt angles of 8°, 17°, and 17°, respectively. A comparison of each tilt angle under various relative air velocities against the 0° condition shows that all three tilt angles can enhance target deposition and penetration ratio. This indicates that adjusting the tilt angle can offset the effects of side wind on droplet drift. As the relative air velocity increases, the penetration ratio decreases under a 0° tilt angle, highlighting a significant effect of relative air velocity on droplet dynamics. At an 8° tilt angle, the penetration ratio gradually decreases with an increase in relative air velocity, with a peak observed at 1 m/s. At a 17° tilt angle, the penetration ratio first rises and then falls with an increase in relative air velocity, reaching a peak at 2 m/s and showing a 3.2% improvement over the baseline. At a 25° tilt angle, the penetration ratio climbs with increasing relative air velocity, achieving its highest value at 3 m/s, the greatest improvement among all tests at 10.3%, with a 1.5% increase also noted at 2 m/s.

The experimental findings affirm that the optimal tilt angles for the maximum droplet penetration ratios across the three wind speeds are in agreement with theoretical predictions. Compared to spray experiments, the configurations leading to the most significant change in deposition amount were a 23° tilt angle and a 3 m/s side wind velocity, while for field tests, the greatest change in deposition amount was seen at a 17° tilt angle and a 3 m/s relative air velocity. The conditions yielding the highest droplet penetration ratio were a 23° tilt angle and a 3 m/s side wind velocity for laboratory tests and a 25° tilt angle and a 3 m/s relative air velocity for field tests. This analysis of observations from both laboratory and field experiments demonstrates that proper adjustment of the nozzle tilt angle in response to side wind can significantly improve both average deposition and penetration ratio, thereby enhancing droplet penetration effectiveness.

## 4. Discussion

The study investigated the spray penetration patterns of horizontal spraying in the presence of orthogonal crosswinds with various nozzle tilt angles, focusing on analyzing two primary indicators: penetrative deposition quantity and penetration ratio. The following conclusions were drawn from field validation experiments:

- The deposition of droplets on and within the canopy is influenced by lateral crosswinds. Adjusting the nozzle tilt angle against the wind direction effectively enhances the average deposition of droplets across the canopy layers, although this improvement lessens with deeper canopy penetration. Laboratory tests across seven nozzle tilt angles showed the most significant effect on penetrative deposition quantity at a 23° tilt, with an average change in deposition amount of +16.705 μL/cm^2^. The most considerable overall change in deposition amount, 28.284 μL/cm^2^, was observed at this angle under a 3 m/s wind speed;
- The droplet penetration ratio is affected by both wind speed and nozzle tilt angle, impacting the attenuation effect during penetration within the canopy and demonstrating a certain level of correlation. Wind speed exerts a more substantial influence than the tilt angle. Increased wind speeds coupled with augmented nozzle tilt angles can improve the droplet penetration ratio. Optimal laboratory conditions were identified as a 23° tilt angle and a 3 m/s wind speed, resulting in a 12.6% increase in penetration ratio compared to the control. The data suggested that the most effective nozzle tilt angles for enhancing droplet penetration ratios at wind speeds of 1, 2, and 3 m/s are 8°, 17°, and 25°, respectively;
- The synergistic effects of side wind and nozzle tilt angle can significantly mitigate the attenuation of droplets during canopy penetration. Influenced by dynamic shifts in canopy porosity and the angle of droplet incidence, the minimal attenuation effect was noted at a 3 m/s wind speed.

Research indicates that adjusting nozzle tilt angles and accounting for side wind speeds can significantly enhance pesticide penetration in plant protection operations, a finding of substantial significance for global agricultural practices. For instance, integrating droplet prediction models into spraying systems to establish a “digital twin” system could enable real-time adjustment of optimal parameters [30], thereby increasing the efficiency of pesticide use and crop protection outcomes. In environments where resources are limited, reducing pesticide usage not only lowers agricultural production costs but also diminishes environmental impact, thus promoting sustainable agricultural development.

While this study has made progress in certain areas, such as elucidating the impact of side wind speed and nozzle tilt angle on droplet penetration in citrus tree protection sprays and proposing a set of optimized spraying parameters, it also encounters limitations, particularly the lack of examination into long-term effects. Future research directions will encompass conducting long-term field experiments to explore the prolonged impact of optimized spraying parameters in actual agricultural production and investigating how factors such as wind direction changes, leaf density, air humidity, and tractor speed collectively influence spray penetration and efficiency. This will require the comprehensive application of various experimental methods and model simulation techniques to ensure the thoroughness and accuracy of research findings. Through these studies, it is hoped to provide deeper theoretical and practical foundations for further optimization of agricultural spraying technology, especially under the current global environmental challenges and resource constraints, to drive agricultural production toward more efficient and environmentally friendly directions.

## Figures and Tables

**Table 1:** Droplet penetration ratio for comparison tests.

Conditions	P1	P2	P
control group	0.375	0.213	0.294
experimental group	0.390	0.180	0.285

**Table 2:** Average deposition for different parameters.

Nozzle Tilt Angle (°)	Side Wind Speed (m/s)	Value of Change in Deposition D_0_ (μL/cm^2^)	Value of Change in Deposition D_1_ (μL/cm^2^)	Value of Change in Deposition D_2_ (μL/cm^2^)
8	1	3.418	3.652	0.632
	2	5.100	1.337	1.041
	3	6.555	1.256	0.857
15	1	3.089	2.499	0.220
	2	7.116	4.214	2.121
	3	12.985	6.869	4.365
23	1	3.967	1.853	1.007
	2	9.539	3.385	2.080
	3	15.011	8.502	4.771
30	1	3.355	1.384	−0.536
	2	11.671	4.969	0.884
	3	18.322	2.740	0.409
38	1	1.308	0.776	−0.474
	2	10.932	3.089	0.703
	3	12.901	6.100	0.378
45	1	2.955	−1.194	−1.072
	2	12.733	0.545	0.708
	3	12.074	2.806	0.537

**Table 3:** The values of the fitted parameters.

Parameters	Numerical Value
a	0.03047 ± 0.00116
b	−2.75182 × 10^−4^ ± 5.13473 × 10^−6^
c	8.69316 × 10^−4^ ± 6.87885 × 10^−5^
d	−0.10403 ± 0.00391
e	0.00827 ± 2.60412 × 10^−4^
f	0.21782 ± 0.00394
R^2^	0.8522

## Data Availability

Due to the organization’s privacy policy, the data of this study are not publicly available.

## References

[1] Liu X.M., Liu X.H., Cui H.Y., Yuan J. (2021). Progress and Prospect of Research on Crop Canopy Droplet Deposition. J. Agric. Mach..

[2] Lv X.L., Fu X.M., Wu P., Ding S.M., Zhou L.F., Yan H.J. (2011). Experimental Study on the Influence of Spray Technology Parameters on Droplet Deposition Distribution. J. Agric. Mach..

[3] Liu X.H. (2021). Study on the Penetration Law of Wind-Blown Droplets in Closed Canopies Based on Layered Porosity. Ph.D. Thesis.

[4] Sun C.D., Liu C.D. (2019). Construction and Application of a Model for Wind-Blown Spray Droplet Penetration in Canopies. J. Agric. Eng..

[5] Sharpe S.M., Boyd N.S., Dittmar P.J., MacDonald G.E., Darnell R.L., Ferrell J.A. (2018). Spray Penetration into a Strawberry Canopy as Affected by Canopy Structure, Nozzle Type, and Application Volume. Weed Technol..

[6] Emanuele C., Giuseppe M., Rita P., Domenico L. (2021). Modelling Spray Pressure Effects on Droplet Size Distribution from Agricultural Nozzles. Appl. Sci..

[7] Uk S., Courshee R.J. (1982). Distribution and Likely Effectiveness of Spray Deposits within a Cotton Canopy from Fine Ultralow-Volume Spray Applied by Aircraft. Pestic. Sci..

[8] Zhu H., Rowland D.L., Dorner J.W., Derksen R.C., Sorensen R.B. (2002). Influence of Plant Structure, Orifice Size, and Nozzle Inclination on Spray Penetration into Peanut Canopy. Trans. ASAE.

[9] Duga A.T., Ruysen K., Dekeyser D., Nuyttens D., Bylemans D., Nicolai B.M., Verboven P. (2015). Spray Deposition Profiles in Pome Fruit Trees: Effects of Sprayer Design, Training System and Tree Canopy Characteristics. Crop Prot..

[10] Foqué D., Nuyttens D. (2011). Influence of Nozzle Type and Spray Angle on Spray Deposition in Ivy Pot Plants. Pest Manag. Sci..

[11] Ru Y., Hu C., Chen X., Yang F., Zhang C., Li J., Fang S. (2023). A Droplet Penetration Model for Spray Applications Based on Canopy Porosity. Agriculture.

[12] Wei Z., Li R., Xue X., Sun Y., Zhang S., Li Q., Chang C., Zhang Z., Sun Y., Dou Q. (2023). Research Status, Methods, and Prospects of Air-Assisted Spray Technology. Agronomy.

[13] Zhai C.Y., Zhao C.J., Ning W., John L., Wang X., Paul W., Zhang H.H. (2018). Research Progress on Precision Control Methods of Orchard Air-Assisted Spraying. J. Agric. Eng..

[14] Zhang Y., Huang X., Lan Y., Wang L., Lu X., Yan K., Deng J., Zeng W. (2021). Development and Prospect of UAV-Based Aerial Electrostatic Spray Technology in China. Appl. Sci..

[15] Law S.E. (2001). Agricultural Electrostatic Spray Application: A Review of Significant Research and Development During the 20th Century. J. Electrost..

[16] Miller P.C.H., Ellis M.C.B. (2000). Effects of Formulation on Spray Nozzle Performance for Applications from Ground-Based Boom Sprayers. Crop Prot..

[17] Zheng J. (2021). Development and Prospect in Environment-Friendly Pesticide Sprayers. J. Agric. Mach./Trans. Chin. Soc. Agric. Mach..

[18] Sun D.Z., Zhan X.R., Liu W.K., Xue X.Y., Xie J.X., Li Z., Song S.R., Wang W.X. (2021). Compensation for Droplet Drift under Crosswind by Adjusting Spray Head Angle. J. Agric. Eng..

[19] Ferguson J.C., Chechetto R.G., Hewitt A.J., Chauhan B.S., Adkins S.W., Kruger G.R., O’Donnell C.C. (2016). Assessing the Deposition and Canopy Penetration of Nozzles with Different Spray Qualities in an Oat Canopy. Crop Prot..

[20] Legleiter T.R., Johnson W.G. (2016). Herbicide Coverage in Narrow Row Soybean as Influenced by Spray Nozzle Design and Carrier Volume. Crop Prot..

[21] Zhu H., Dorner J.W., Rowland D.L., Derksen R.C., Ozkan H.E. (2004). Spray Penetration into Peanut Canopies with Hydraulic Nozzle Tips. Biosyst. Eng..

[22] Salyani M., Zhu H., Sweeb R.D., Pai N. (2013). Assessment of Spray Distribution with Water-Sensitive Paper. Agric. Eng. Int. CIGR J..

[23] Ovidiu R., Ovidiu M., Valentin M.M., Adrian M., Bogdan G.A., Valentin C., Sorin S., Tibor R. (2021). Quality Analysis of Some Spray Parameters When Performing Treatments in Vineyards in Order to Reduce Environment Pollution. Sustainability.

[24] Liu X.D., Xu Y., Hu C.X., Gu Y.W., Zhou J.P., Feng Z.J. (2024). Study on Droplet Deposition Characteristics of Heavy-Duty Plant Protection UAV Based on Fluent. Agric. Mech. Res..

[25] Wang L., Lan Y.B., Hoffmann W.C., Fritz B.K., Chen D., Wang S.M. (2016). Design and Droplet Deposition Pattern Study of a Micro Unmanned Aerial Vehicle (UAV) Low-Altitude Variable Spraying System. J. Agric. Mach..

[26] Zhang H.C., Zheng J.Q., Zhou H.P., Dorr G.J. (2017). Study on Droplet Deposition Distribution and Off-Target Drift During Pesticide Application. J. Agric. Mach..

[27] Shen L., Fang G., Wang S., Xing F., Chan S. (2022). Numerical Study of the Secondary Atomization Characteristics and Droplet Distribution of Pressure Swirl Atomizers. Fuel.

[28] Liu X.M., Song L.Q., Cui H.Y., Liu Y.C., Liu X.H., Wu M.Q. (2021). Decoupling Study on the Impact of Airflow Droplet Stress and Canopy Porosity Changes on Deposition Performance. J. Agric. Mach..

[29] Shang Q.Q., Zhang Y.Q., Sun Z.W., Zheng J.D., Zhao B.G. (2004). Study on Droplet Deposition and Penetration in Tree Canopies. J. Nanjing For. Univ. (Nat. Sci. Ed.).

[30] Zhou R.Q., Zhang H.C., Zheng J.Q., Zhou H.P., Tang Y.S., Wang D. (2019). Analysis of the Impact of Mobile Spraying Parameters on Droplet Penetration for Forest Pest and Disease Control. J. Cent. South Univ. For. Technol..

